# Priming with proangiogenic growth factors and endothelial progenitor cells improves revascularization in linear diabetic wounds

**DOI:** 10.3892/ijmm.2014.1630

**Published:** 2014-01-21

**Authors:** MAXIMILIAN ACKERMANN, ANDREAS M. PABST, JAN P. HOUDEK, THOMAS ZIEBART, MORITZ A. KONERDING

**Affiliations:** 1 Institute of Functional and Clinical Anatomy, University Medical Center Mainz, D-55131 Mainz, Germany; 2 Department of Oral and Maxillofacial Surgery, University Medical Center Mainz, D-55131 Mainz, Germany

**Keywords:** priming, proangiogenic, endothelial progenitor cells, wound healing, diabetic mice

## Abstract

In the present study, we investigated whether proangiogenic growth factors and endothelial progenitor cells (EPCs) induce favourable effects on cutaneous incisional wound healing in diabetic mice. The proangiogenic effects of human EPCs were initially analyzed using a HUVEC *in vitro* angiogenesis assay and an *in vivo* Matrigel assay in nude mice (n=12). For the diabetic wound model, 48 Balb/c mice with streptozotocin (STZ)-induced diabetes were divided randomly into 4 groups (12 mice in each group). Subsequently, 3, 5 and 7 days before a 15-mm full-thickness incisional skin wound was set, group 1 was pre-treated subcutaneously with a mixture of vascular endothelial growth factor (VEGF)/basic fibroblast growth factor (bFGF)/platelet-derived growth factor (PDGF) (3.5 μg of each), group 2 with 3.5 μg PDGF and group 3 with an aliquot of two million EPCs, whereas the control animals (group 4) were pre-treated with 0.2 ml saline solution. The wounds were assessed daily and the repaired tissues were harvested 7 days after complete wound closure. The angiogenesis assay demonstrated significantly increased sprout densities, areas and lengths in the EPC-treated group (all p<0.01). In the Matrigel assay, significantly increased microvessel densities, areas and sizes (all p<0.001) were also detected in the EPC-treated group. In the STZ-induced model of diabetes, the animals pre-treated with a combination of proangiogenic factors and EPCs showed in general, a more rapid wound closure. Vessel densities were >2-fold higher in the mice treated with a combination of proangiogenic factors and EPCs (p<0.05) and tensile strengths were higher in the groups treated with proangiogenic growth factors compared to the controls (p<0.05). These results suggest a beneficial effect of pre-treatment with proangiogenic growth factors and EPCs in incisional wound healing.

## Introduction

Diabetes affects approximately 170 million individuals worldwide, and it is anticipated that this number will double over the next 20 years (1). The diminished production of proangiogenic growth factors and decreased wound angiogenesis are thought to contribute to impaired wound repair in diabetic patients (2), resulting in scarring, delayed and compromised mechanical wound healing (3). Wound healing is strongly dependent on the formation of granulation tissue, which in turn intimately correlates with the induction of new vessel formation. Wound angiogenesis represents a merged process that relies on the extracellular matrix in the wound bed, as well as on the migration and mitogenic stimulation of endothelial cells (4). Therefore, several studies have investigated whether the stimulation of angiogenesis can promote diabetic wound healing (5).

Adenoviral gene transfer with proangiogenic growth factors [particularly vascular endothelial growth factor (VEGF)] has been shown to accelerate diabetic wound healing, whereas the topical application with VEGF or platelet-derived growth factor (PDGF) has shown ambivalent results (6). Our own previous experiments have demonstrated the potential therapeutic benefits of priming with proangiogenic factors and cell prior to surgery with a combination of proangiogenic growth factors for wound healing in normoglycemic and diabetic mice (7,8). Priming with a combination of supraphysiological doses of VEGF, fibroblast growth factor (FGF) and PDGF led to more rapid times to closure, higher vessel densities and better functional outcomes (8). In addition to proangiogenic growth factors, endothelial progenitor cells (EPCs) may present a potential pre-treatment option for diabetic wounds.

Asahara *et al* identified circulating EPCs as the key cell type contributing to neovascularization (9), and subsequent studies revealed that bone marrow (BM)-derived EPCs are essential for the tissue repair process in ischemia-induced damage of the limbs, kidneys and heart (10–12). BM-derived EPCs can also be incorporated into newly formed capillaries in granulation tissue, thereby promoting neovascularization during wound healing (13,14).

EPCs may be incorporated into newly formed vessels through multiple steps, including sensing the ischemic signal from the remote tissue, releasing EPCs from the BM niche into the circulation, homing circulating EPCs to the target tissues, integrating EPCs into blood vessels and the *in situ* differentiation/maturation of EPCs into mature and functional endothelial cells (15,16). Tissue ischemia is presumed to be the strongest stimulus for EPC mobilization from the BM to the circulation (11,17). Moreover, EPC mobilization can be augmented by various cytokines, including granulocyte colony-stimulating factor (G-CSF), granulocyte-macrophage colony-stimulating factor (GM-CSF), VEGF and placental growth factor (PGF) (18–21). With regard to the fact that EPCs provide both, strong autocrine and paracrine proangiogenic effects, as well as the building material for vessel creation, EPCs may present a powerful treatment option to improve revascularization and wound healing in diabetic wounds.

The aim of this study was to eludicate the *in vitro* and *in vivo* effects of pre-treatment with a combination of proangiogenic growth factors (VEGF, bFGF and PDGF), a monotherapy with PDGF and with EPCs on the healing of diabetic incisional wounds.

## Materials and methods

### EPC isolation and culture

Mononuclear cells (MNCs) were isolated by density gradient centrifugation with Biocoll (Biochrom KG, Berlin, Germany) from peripheral human blood as previously described (21). Immediately following isolation, total MNCs (8×10^6^ cells/ml medium) were plated on 25 cm^2^ culture flasks coated with human fibronectin (Sigma, Steinheim, Germany) and maintained in endothelial basal medium (EBM) supplemented with EGM SingleQuots, 100 ng/ml VEGF and 20% fetal calf serum (FCS).

### In vitro angiogenesis assay

To evaluate the proangiogenic potential of EPCs *in vitro*, a commercial HUVEC angiogenesis assay (3D Angiogenesis Assay; PromoCell, Heidelberg, Germany) was used. The assay was performed according to the manufacturer’s instructions. EPCs were added to 8 of the 16 wells (2×10^6^ cells/well, dissolved in 400 μl EPC culture medium). The remaining 8 wells served as controls. The sprouting colonies were photo-documented over a period of 48 h. For the measurement of sprout densities, areas and lengths, a software program (CellAnalyzer 2.25) was used as previously described (23).

### Animals

Twelve nude mice (obtained from Charles River Laboratories Inc., Wilmington, MA, USA), 25–28 g, were used for the *in vivo* Matrigel assay. Forty-eight female Balb/c mice, 25–33 g, were used for the diabetic wound experiments, which were obtained from the Central Animal Facilities of the University of Mainz, Mainz, Germany. All mice were allowed to acclimate for 14 days prior to the treatment and were housed in an approved animal care facility with 12-h light cycles. Food and water were provided *ad libitum*. The care of the animals was consistent with the legal guidelines and all experiments were conducted after obtaining approval from the local animal welfare authorities (licence no. 1.5 177-07/041-2). Animals were kept in individual cages during the experiment in order to avoid biting and interference with the wounds. Soft tissue paper was used instead of conventional bedding in order to avoid wound irritation or contamination.

### In vivo Matrigel assay

Twelve nude mice were divided randomly into 2 groups (6 mice in each group). The mice were anesthetized with an intraperitoneal injection of avertin [1.5 ml/100 g body weight (BW), 2,2,2-tribromoethanol; Sigma-Aldrich, Munich, Germany] and a total of 500 μl of Matrigel Basement Membrane Matrix (BD Biosciences, Franklin Lakes, NJ, USA) containing 100 ng/ml VEGF (PeproTech, Rocky Hill, NJ, USA) and 100 U/ml of heparin (Sigma, St. Louis, MO, USA) was injected subcutaneously in the dorsal midline of the 12 mice, using a 26-gauge needle. Group I mice were administered a retro-orbital, intravenous injection of EPCs (2×10^6^ cells), dissolved in 100 μl phosphate-buffered saline (PBS) per animal. Group II mice were administered a retro-orbital, intravenous injection of 100 μl PBS as the controls. After 2 weeks, 5 mice from each group were sacrificed by cervical dislocation and the Matrigel plugs were removed and prepared for histological analysis and immunochemistry. The remaining 2 mice (1 from each group) were used for microvascular corrosion casting. A previous study demonstrated that 2 weeks are a sufficient time period to allow measurable angiogenesis in the implanted Matrigel plugs (22).

### Model of streptozotocin (STZ)-induced diabetes

To assess the effects of priming on diabetic mice, hyperglycaemia was induced by intraperitoneal injections (24) of 150 mg/kg BW STZ (Sigma-Aldrich, Taufkirchen, Germany). The injection of STZ was repeated twice every other day with a concentration of 50 mg/kg BW. Blood glucose levels were determined weekly and mice that did not show hyperglycaemia ≥250 mg/dl at the time of surgery were excluded from the study.

### Proangiogenic priming

Forty-eight Balb/c mice with STZ-induced diabetes were divided randomly into 4 groups (12 mice in each group). Prior to the proangiogenic priming, the dorsal skin of the mice was shaved and depilated. Subsequently, 7, 5 and 3 days prior to surgery, group 1 was pre-treated with a mixture of VEGF (35.0 μg; R&D Systems, Minneapolis, MN, USA), bFGF (2.5 μg; R&D Systems) and PDGF (3.5 μg; R&D Systems). Group 2 was pre-treated with PDGF (3.5 μg; R&D Systems) and group 3 with an aliquot of two million EPCs. Proangiogenic growth factors and EPCs were particulary dissolved in 0.2 ml saline solution (B. Braun Melsungen AG, Melsungen, Germany). The control group (group 4 was pre-treated with 0.2 ml pure saline solution. The injections were administered subcutaneously 1.0 cm paramedian under the dorsal skin of the mice after skin disinfection with 70% ethanol.

### Surgical procedure

The mice were anesthetized with an intraperitoneal injection of avertin (1.5 ml/100 g BW, 2,2,2-tribromoethanol; Sigma-Aldrich, Munich, Germany). Immediately prior to surgery, an area of 15×15 mm was shaved again using disposable shavers followed by skin disinfections with 70% ethanol. Under sterile conditions, 15-mm-long full-thickness incisional skin wounds were set in the midline of the lumbar dorsal skin of the 48 mice and closed by 4 single button sutures using absorbable suture material (Vicryl 4-0; Ethicon, Hamburg, Germany).

### Tissue sampling

#### In vivo Matrigel assay

The central parts of the removed Matrigel plugs were used for histological analysis [hematoxylin and eosin (H&E)] and immunochemistry (CD31) according to standard protocols. The CD31 stained sections were photographed (Leica MZ12) and discretely analyzed by a Weibel Grid analysis system for exact quantification (25). Microvessel density (MVD) was expressed as the number of microvessels (MV) per square millimeter. The microvessel area (MVA) was expressed as the percentage area of all microvessels in a section proportional to the section Matrigel-area and microvessel size (MVS) was expressed as the mean size of the vessels. Additionally, 1 mouse from the control group and 1 mouse from the EPC-treated group was used for microvascular corrosion casting as previously described (26).

#### Model STZ-induced diabetes

Animals in all groups were sacrificed on day 14 and 21 after surgery. The central parts of the wound area were used for the maximum tensile strength measurements, and the adjacent parts for histological analysis and immunochemistry. Five-micrometer-thin sections of paraffin-embedded specimens were stained with H&E according to standard protocols. H&E-stained sections were used to assess tissue layer thicknesses of the epidermis and dermis, scar thickness and the progress of remodeling with image analyzing software Diskus 4.80 (Hilgers, Königswinter, Germany). Immunohistochemical staining of endothelial cells was performed using a monoclonal antibody against CD31 (BD Biosciences Pharmingen, Heidelberg, Germany). Antibody binding was visualized via a 3-step staining procedure using a biotinylated polyclonal anti rat IgG secondary antibody (DakoCytomation GmbH, Hamburg, Germany) and the streptavidin horseradish peroxidase reaction together with the DAB detection system. Vessel densities and lymphatic densities were assessed using a Weibel grid, as previously described (25) and expressed as a percentage of the surface area.

#### Tensile strength measurements

Test strips were punched out from the harvested wound vertically to the craniocaudal axis on day 24 post-surgery. The test strips had a defined hour glass form with 3-mm width at the narrowest part constituting a pre-determined breaking point. The design of the hour glass form was based on material testing standards. Test strips without the hour glass form would inevitably tear at the wedge grips, where the tissue is already bruised. The breaking strength test device consisted of 2 opposing gripping jaws which fixed the tissue strip. The electric motor driven gripping jaws were moved apart with a constant strain rate of 0.5 mm/sec under displacement control. Time, force and displacement were recorded for stretching up until failure. A position encoder (WA300) was used to register the stretching distance; a force transducer (S2, maximum value 150 N) was used to quantify the power applied to the tissue strip. The endpoint was the breaking strength in Newton (kg·m/sec^2^). The resulting values were recorded by a multiple channel PC measuring device [Spider 8, Hottinger Baldwin Messtechnik GmbH (HBM), Darmstadt, Germany] and plotted as a force-deflection curve (software, Catman 4.5, all from HBM). The maximum breaking strength was determined from the stress-strain curve.

#### Statistical analysis

Statistical analysis was based on measurements in at least 34 different mice. The unpaired Student’s t-test for samples of unequal variances was used to calculate statistical significance. The data are expressed as the means ± one standard deviation. The significance level for the sample distribution was defined as p<0.05.

## Results

### In vitro angiogenesis assay

**Figure 1:**
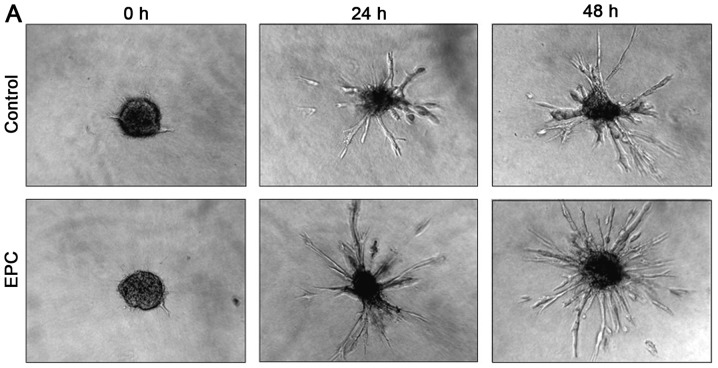

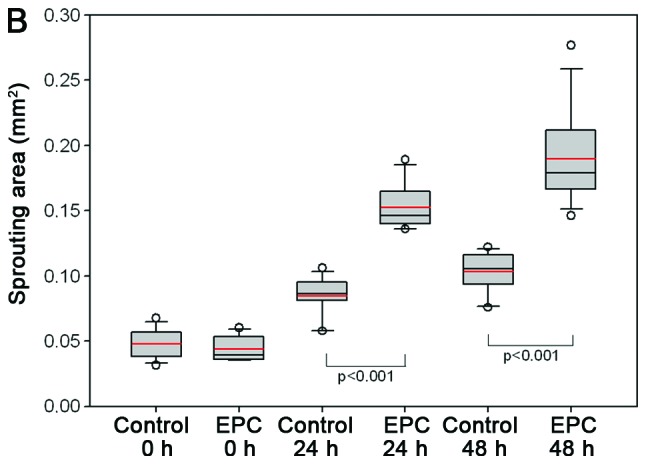
*In vitro* angiogenesis assay. (A)The group treated with endothelial progenitor cells (EPCs) revealed markedly increased sprout densities and lengths after 24 and 48 h in comparison to the control group. (B) Quantification of sprouting showed significantly higher sprouting areas in the EPC-treated wells. Box-whisker plots showing the median, 5th, 10th, 25th, 75th, 90th and 95th percentile.

Fig. 1A shows exemplary images of HUVEC sprouts directly after EPC incubation and after 24 and 48 h. The control group presented a dense, radial sprouting out of the initial cell spheroid. In comparison to the control group (p_0 h_=0.0479 mm^2^, p_24 h_=0.0848 mm^2^, p_48 h_=0.1034 mm^2^), incubation with EPCs demonstrated significant increased sprouting areas (p_0 h_=0.0438 mm^2^, p_24 h_=0.1523 mm^2^, p_48 h_=0.1899 mm^2^) (p<0.001) (Fig. 1B).

### In vivo Matrigel assay

**Figure 2:**
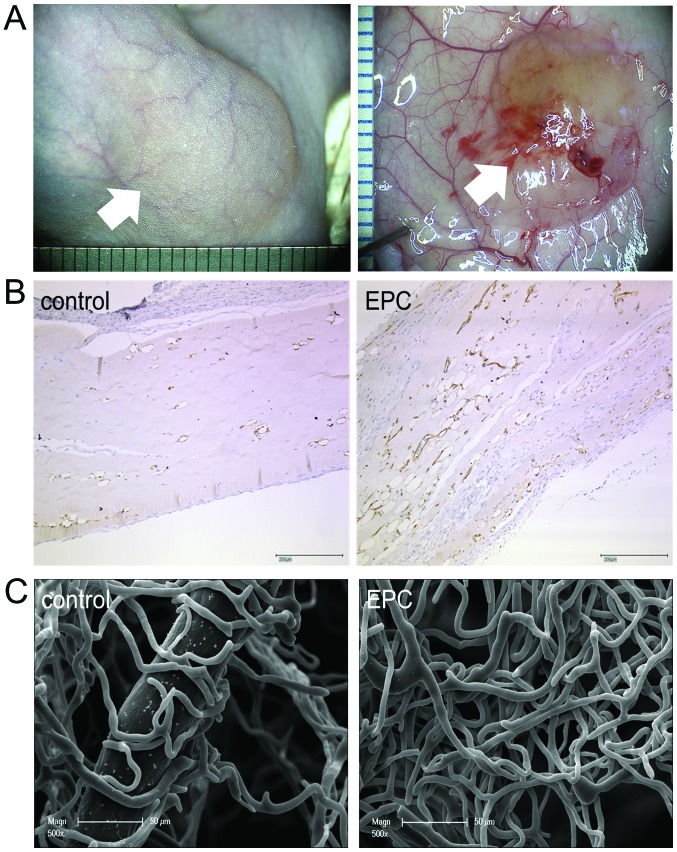
*In vivo* Matrigel assay. (A) A Matrigel plug was implanted subcutaneously under the dorsal skin of nude mice *in situ* (left panel, white arrow). (B) Explanted Matrigel plug after 14 days (right panel, white arrow). (C) Scanning electron microscope (SEM) scans of the microvascular corrosion casts [control group, left panel; endothelial progenitor cell (EPC)-treated group, right panel] show the higher vascular density in the EPC-treated animals.

After the explantation of the Matrigel plug (Fig. 2A), in the anti-CD31-stained Matrigel sections (Fig. 2B) and in the microvascular corrosion casts (Fig. 2C), a higher vascular density in the EPC-treated plugs compared to the controls was observed. Morphometrical analysis revealed that the EPC application increased the MVD significantly up to 135.1±32.2 MV/mm^2^ (p<0.001) compared to the control group with a median of 64.9±5.0 MV/mm^2^. The EPC-pre-treated animals showed an MVA of 5.4±1.6%, whereas the control group showed an MVA of 2.0±0.1%. The EPC application increased the MVS significantly up to a median MVS of 402.3±30.8 μm^2^ (p<0.0001), whereas the control group had a median MVS of 309.1±18.1 μm^2^.

### Gross appearance

**Figure 3:**
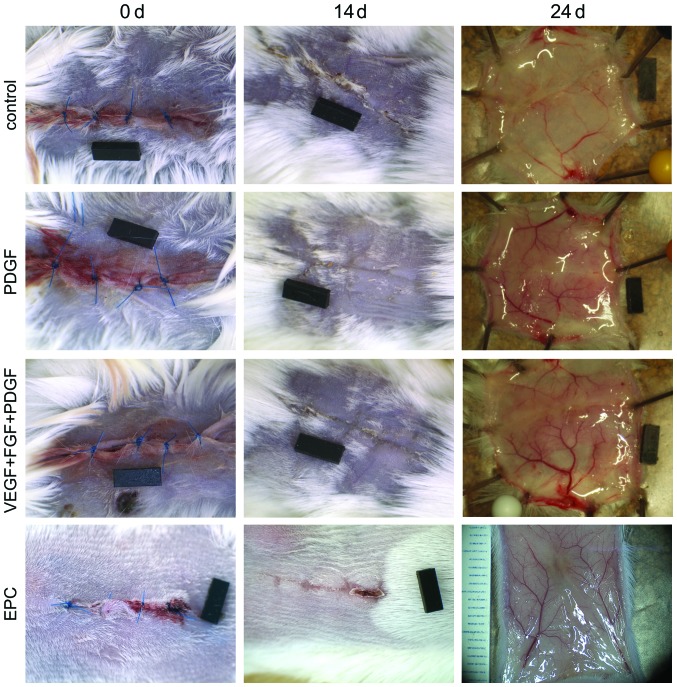
Gross appearance. Macroscopic assessment of wound closure on day 14 and 24 (backside of specimen biopsy) in representative mice receiving either control treatment (0.2 ml isotonic saline solution), a combination of proangiogenic growth factors (a combination of 35.0 μg VEGF, 2.5 μg bFGF and 3.5 μg PDGF), PDGF-monotherapy (3.5 μg PDGF), or endothelial progenitor cells (EPCs) (2 million cells). The black marker has a length of 5 mm. d, days.

As shown in Fig. 3, the wounds of all the animals were correctly adapted on day 14 and 24 after surgery. Macroscopically, a more rapid wound closure was observed in the treated animals. The higher vessel densities of the groups pre-treated with proangiogenic growth factors were evident on day 24 after harvesting.

### Vessel density

**Figure 4:**
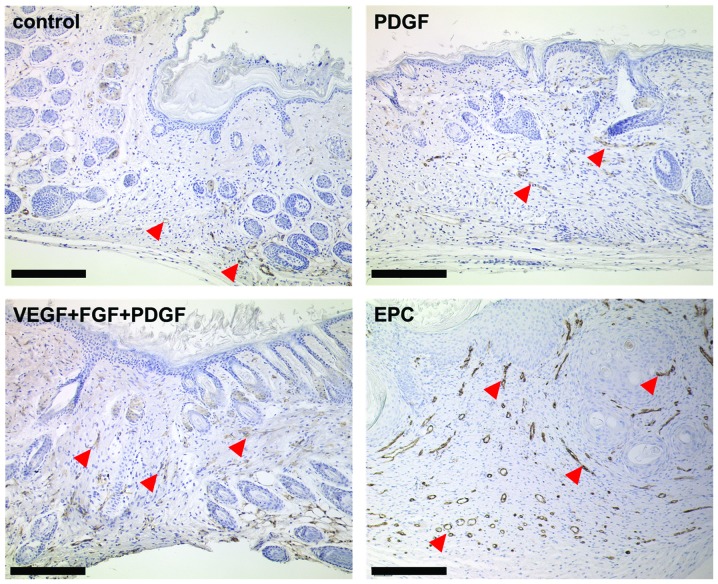
Vascular morphology. Vascularization of excised wound biopsies from control animals (0.2 ml isotonic saline solution), or animals treated with PDGF monotherapy (3.5 μg), a combination of proangiogenic growth factors (35.0μg VEGF, 2.5 μg bFGF and 3.5 μg PDGF), and endothelial progenitor cells (EPCs) (2 million cells) 14 days after wounding. Note the higher vessel density (red arrowheads), cell invasion and higher re-epithelialization in the EPC- and proangiogenic combination-primed animals. Bars, 200 μm.

**Figure 5:**
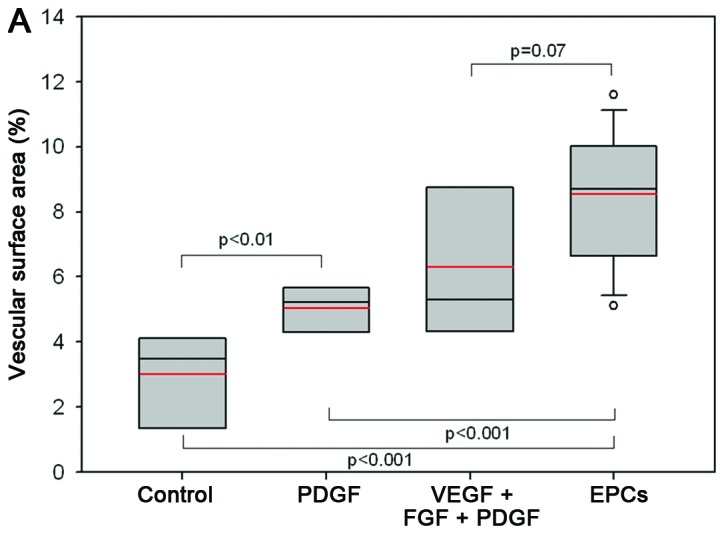

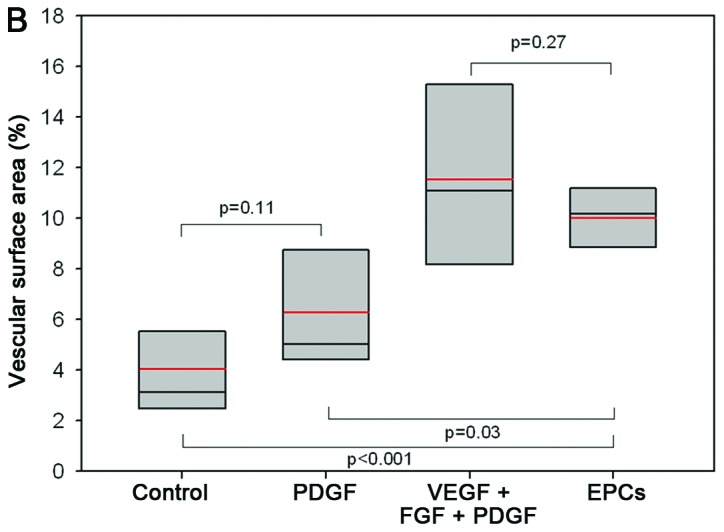
(A) Microvessel densities of harvested wound tissue from the control animals (0,2 ml isotonic saline solution), or those treated with PDGF monotherapy (3.5 μg PDGF), a combination of proangiogenic growth factors (a combination of 35.0 μg VEGF, 2.5μg bFGF and 3.5μg PDGF), or endothelial progenitor cells (EPCs) (2 million cells) on (A) day 14 and (B) day 24 after wounding. Box-whisker plot showing the median, 10th, 25th, 75th and 90th percentile and mean (red).

The vessel densities were determined in the wounds after sacrificing the animals on day 14 and 24 after surgery. Assessment of the local vessel expression revealed that vessel allocation accumulated in particular in the subcutis and panniculus carnosus (Fig. 4). The harvested tissue of the animals primed with a combination of proangiogenic growth factors showed a higher cell density that invaded the dermis (Fig. 4). Fig. 5A shows that priming resulted in significantly higher percentual vessel densities 14 days after wounding: priming yielded >2-fold higher vessel surface areas in the groups primed with a combination of proangiogenic growth factors and EPCs vs. the controls (mean, 4.5 vs. 2.8% vascular surface area; p<0.01). Following complete wound closure and harvesting, vessel densities were assessed separately in the wound ground, the former wound margin and in the adjacent unwounded tissue. Fig. 5B illustrates that the differences in vessel densities occurred on day 24 post-surgery. The animals primed with a combination of proangiogenic growth factors (VEGF + FGF + PDGF) yielded vessel densities of 11.5±3.5% (mean), the EPC-pre-treated mice densities of 10.0±1.4%, whereas the PDGF-primed mice (6.3±3.2%) and the controls (4.0±2.3%) revealed significant lower vessel densities (p<0.05). Nonetheless, the highest values were observed in the pre-treated animals.

### Maximum tensile strength

**Figure 6:**
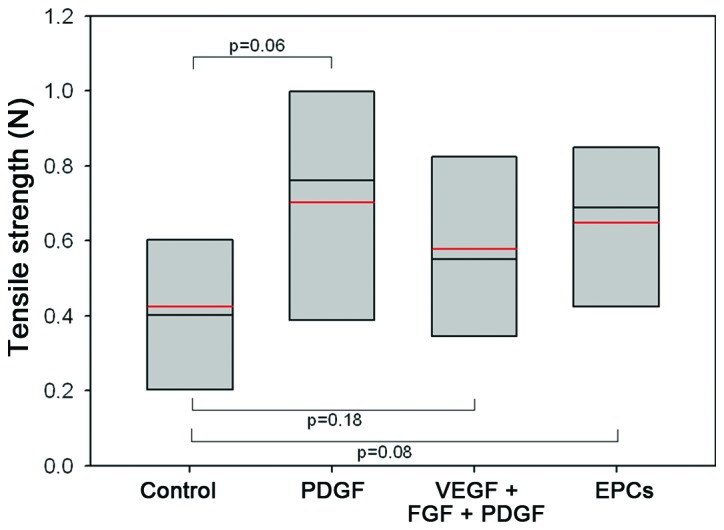
Comparison of tensile strength values obtained on day 24 after surgery. Mice received either control treatment (0.2 ml isotonic saline solution), PDGF monotherapy (3.5 μg), combination treatment (35.0 μg VEGF, 2.5 μg bFGF, and 3.5 μg PDGF), or endothelial progenitor cells (EPCs) (2 million cells) on days 3, 5 and 7 prior to surgery. Box-whisker plot showing the median, 10th, 25th, 75th, and 90th percentile and mean (red).

Functional tensile strength testing revealed a certain advantage of the group pre-treated with proangiogenic growth factors in comparison to the sham-treated control group; however, significant differences were not observed (Fig. 6). The mean values of the animals primed with a combination of proangiogenic growth factors wer 0.58±0.28 N. The PDGF-monotherapy group showed the highest values (0.70±0.33 N) and the control animals showed a value of 0.43±0.23 N. The animals pre-treated with EPCs revealed a mean tensile strength of 0.65±0.24 N.

## Discussion

This study demonstrates that priming with proangiogenic growth factors and EPCs enhances incisional wound healing, as defined by a more rapid wound re-epithelialization, higher wound vascularization and higher tensile strength. In particular, the assessment of time-to-closure and functional outcome revealed an advantage for the groups primed with EPCs in comparison to the control animals. Therefore, the findings in this study allow the presumption that local EPC pre-treatment may be a novel and clinically applicable strategy for enhancing wound healing in diabetic wounds.

A number of studies have reviewed the therapeutic implication of different proangiogenic growth factors or EPCs. In general, the mode of application (topical, systemic, or priming) and the dosage seem to represent variables with the highest impact in terms of boosting angiogenesis in wound healing (27). Whereas topically applied growth factors have been shown to be ineffective (28), the direct application of angiogenic factors, such as VEGF, FGF and PDGF (8), has shown improved angiogenesis and increased functional quality. Sander *et al* demonstrated the positive impact of systemic transplantation of EPCs in mouse ear model (29).

Cell-based therapy is a promising therapeutic option for treating patients with diabetic, non-healing wounds. Of various different types of stem or progenitor cells, the EPC is a type of cell that has been moved from experimental models to clinical trials. A few properties, such as its endogenous, BM-derived characteristics, the ability to home to sites of pathological entities and relative stability in terms of lineage specification in culture, which allows genetic and epigenetic manipulation, make EPCs an ideal cell candidate to be tested in cell-based therapeutic applications for ischemic disorders, such as diabetes. Several mechanisms are impaired in diabetes, the level of circulating EPCs in diabetic wounds is decreased and mobilization is reduced (30,31). Thus, a combination of therapeutic approaches to target individual impairments and to correct diabetes-related EPC deficits will likely synergize and may lead to a more-successful treatment outcome for diabetic wounds.

Taken together, the results from the present study demonstrate that priming with proangiogenic growth factors and EPCs results in more rapid wound closure, higher vessel density and a better functional outcome. Future experiments are planned which will examine the effects of a combination of EPCs and proangiogenic growth factors and compare the effects of proangiogenic EPC priming with gene therapeutic approaches.
